# Surgical Management of Extrafollicular Variant of Adenomatoid Odontogenic Tumor in the Maxillary Posterior Region

**DOI:** 10.1155/2019/3787696

**Published:** 2019-01-29

**Authors:** Gerardo La Monaca, Nicola Pranno, Cira Rosaria Tiziana di Gioia, Giorgio Pompa, Iole Vozza, Maria Paola Cristalli

**Affiliations:** ^1^Department of Sense Organs, Sapienza University of Rome, Rome, Italy; ^2^Department of Oral and Maxillo-Facial Sciences, Sapienza University of Rome, Rome, Italy; ^3^Department of Radiological, Oncological and Pathological Sciences, Sapienza University of Rome, Rome, Italy; ^4^Department of Biotechnologies and Medical Surgical Sciences, Sapienza University of Rome, Rome, Italy

## Abstract

**Background:**

Adenomatoid odontogenic tumor (AOT) is a relatively uncommon benign neoplasm of odontogenic epithelial origin, accounting for less than 5% of odontogenic tumors.

**Case Report:**

The reported case describes morphological characteristics, clinical course, radiographic and histopathological features, and surgical therapy of an extrafollicular variant of AOT in the maxillary posterior region. An asymptomatic swelling on the left side in the posterior region of the maxilla, gradually increased since approximately 12 months, developed in a 16-year-old Caucasian female patient. Radiographic images revealed a well-defined, unilocular radiolucency, with some small foci of radiopacity inside, and root resorption of the first and second molars. On the base of the histological examination of the specimen retrieved by incisional biopsy, the diagnosis of AOT was made, and the conservative surgical enucleation of the lesion was performed.

**Discussion:**

The present case was reported in agreement with an extensive review, in which it was recommended to discontinue reporting classic follicular cases because their clinicopathological profile was well-known, but to continue reporting well-documented cases of the extrafollicular variant, with indication of the exact position.

**Conclusion:**

The present case was reported in order to expand the knowledge about the clinical behavior and surgical treatment of the extrafollicular variant of AOT.

## 1. Introduction

Adenomatoid odontogenic tumor (AOT) is a relatively uncommon benign neoplasm of odontogenic epithelial origin, accounting for less than 5% of odontogenic tumors [1–3].

The currently used name of “adenomatoid odontogenic tumor” (AOT) was proposed by Philipsen et al. [2] in 1969 and adopted for the first time in the1971 first edition of the World Health Organization (WHO) classification of histological typing of odontogenic tumor, jaw cysts, and allied lesion and retained in the 1992 second edition [4].

Later in the WHO third edition (2005) of Head and Neck Tumors, AOT was defined as an odontogenic tumor “composed of odontogenic epithelium in a variety of histoarchitectural patterns, embedded in a mature connective tissue stroma, and characterized by slow but progressive growth” [5]. Recently, in the WHO 4th edition (2017) of Head and Neck Tumors, AOT was defined as “a benign epithelial tumor that shows duct-like structures” [1].

The reported case describes morphological characteristics, clinical course, radiographic and histopathological features, and surgical therapy of an extrafollicular AOT, which developed in the maxillary posterior region of a 16-year-old Caucasian female patient.

## 2. Case Presentation

A young patient (female, 16 years old) was referred to the Oral Surgery Unit of the Policlinico Umberto I Hospital–Sapienza University of Rome with the chief complaint of asymptomatic swelling in the left side in the posterior region of the maxilla, gradually increased to the present size of 3.5 cm since approximately 12 months.

Medical history and extraoral examination were noncontributory, and there was no regional lymphadenopathy.

**Figure 1:**
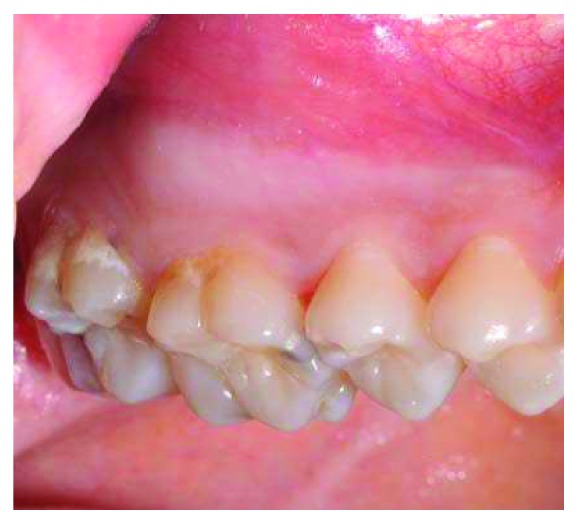
Intraoral view showing a swelling in the buccal fold of the left maxillary posterior region.

Intraoral examination revealed, in the buccal fold of the left maxillary posterior region, a swelling extending from behind the canine up to the tuberosity, covered by normal oral mucosa (Figure 1). On palpation, the buccal cortical plate was expanded, and the swelling was smooth, nontender, and nonfluctuant, and its consistency was bony hard.

The involved teeth were sound, positive at cold sensitivity test, and without mobility.

**Figure 2:**
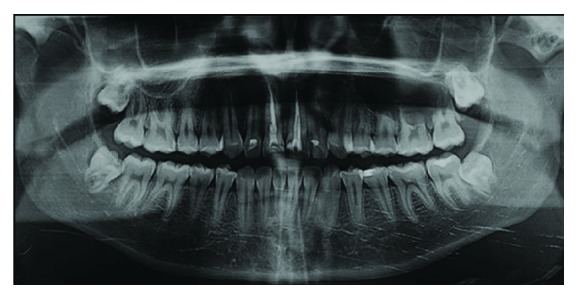
Panoramic radiograph showing in the left maxillary posterior region a radiolucent, unilocular lesion, resorptions of 2.6 roots and 2.7 mesial root, and presence of unerupted third molar.

Panoramic radiograph showed in the left maxillary posterior region a well-defined, unilocular radiolucency, root resorption of the first and second molars, and presence of an unerupted third molar (Figure 2).

**Figure 3:**
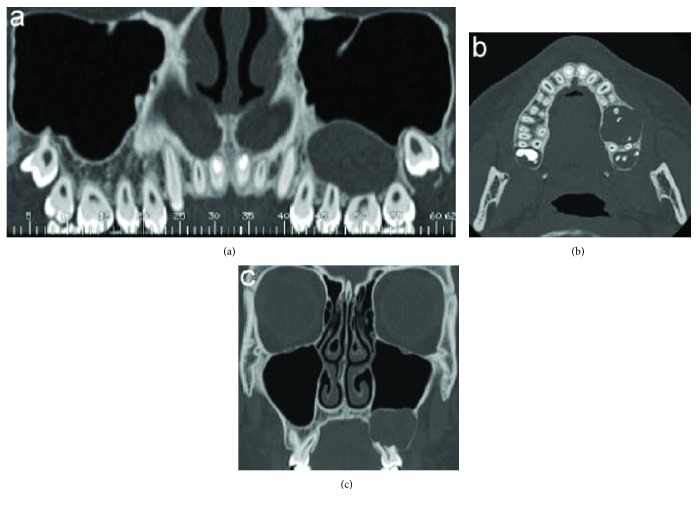
Computed tomography scan: (a) panorex view: unilocular, well defined, hypodense area extending from the mesial root of 2.4 up to the distal root of 2.7 and in the apicocoronal direction from the sinus floor to the alveolar ridge; (b) axial view: limited expansion and thinning of the buccal and palatal cortical plates, erosion of the buccal wall, and some small foci of radiopacity; and (c) coronal view: root resorption of the first molar.

Panorex view of the Computed Tomography (CT) revealed a hypodense intrabony, unilocular lesion circumscribed by radiopaque border, extending from the mesial margin of the first premolar to the distal margin of the second molar and apicocoronally from the sinus floor to the alveolar ridge. The resorption of the first molar roots and the second molar mesial root and the unerupted third molar not related to the lesion were also detectable (Figure 3(a)).

In the axial view of the CT, a limited expansion and thinning of the buccal and palatal cortical plates, limited cortical perforation in the vestibular wall upper the first molar, and small foci of radiopacity near the mesial root of the first molar were observed (Figure 3(b)).

The resorption of the first molar roots was also evident in the coronal view of the CT (Figure 3(c)).

Based on the clinical and radiographic findings, different pathologic conditions, such as dentigerous cyst, calcifying odontogenic cyst, odontogenic keratocyst, central giant cell granuloma, unicystic ameloblastoma, calcifying epithelial odontogenic tumor, ameloblastic fibroma, and ameloblastic fibroodontoma, were considered, and the preventive histological diagnosis was needed for treatment planning.

**Figure 4:**
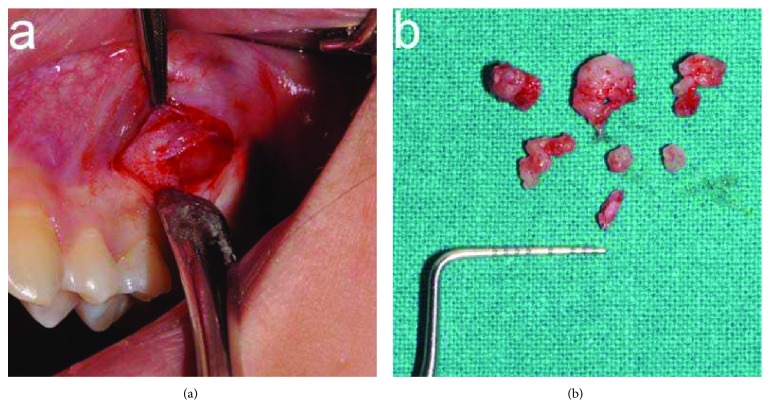
Incisional biopsy: (a) incision in the buccal fold and (b) retrieved specimen.

Incisional biopsy was performed under local anesthesia (Figure 4).

**Figure 5:**
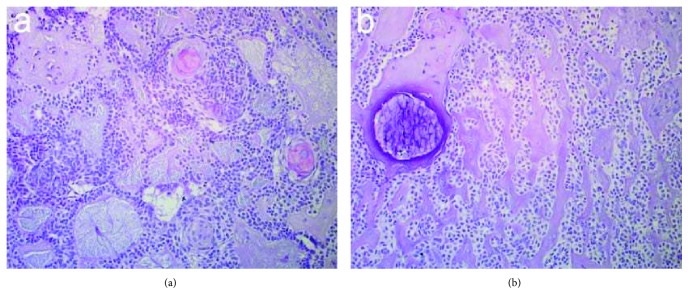
Histopathological sections of incisional biopsy specimen: (a) characteristic of nodular epithelial proliferation composed of polyhedral to spindle cells with lobular or syncytial arrangement of cells (hematoxylin and eosin, 20X) and (b) foci of PAS-positive material and some.

Histological examination showed a nodular proliferation of polyhedral epithelial cells of probable odontogenic origin, organized either in small cystic spaces with intraluminal basophilic PAS-positive material or in syncytial and trabecular nests. The stroma was poorly cellular, either intensely eosinophilic or amorphous like dentin, with some calcifications (Figure 5). A diagnosis of AOT was made.

Conservative surgical enucleation of the lesion, extraction of the first molar, and apicectomy of the involved teeth, previously endodontically-treated, were planned.

**Figure 6:**
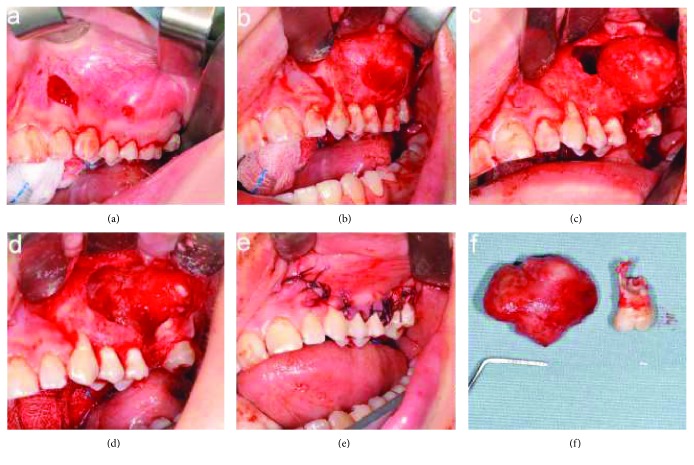
Surgical treatment: (a) a full-thickness intrasulcular trapezoidal buccal flap, (b) detaching of the flap, (c) ostectomy and dissection of the lesion from the bony walls, (d) bony cavity, (e) suture, and (f) surgical specimen.

The surgery was performed under general anesthesia. Surgical access was obtained through a full-thickness trapezoidal intrasulcular buccal flap, extending from the mesial aspect of the canine to the distal aspect of the second molar (Figure 6(a)). The flap was detached, and the cortical bone was exposed (Figure 6(b)).

After the first molar extraction, ostectomy was performed under sterile saline irrigation with a round tungsten carbide burr mounted on a low-speed handpiece used with a tangential movement not to involve the underlying lesion. The fibrotic capsule surrounding the tumor was dissected from the bony wall, and the mass was completely enucleated (Figures 6(c) and 6(d)).

Before flap repositioning, amputation of the resorbed mesial root of the second molar, and apicectomy of the premolars and the remaining roots of the second molar, thorough debridement of the bony cavity and trimming of the rough bony edges were carried out.

A releasing periosteal incision was made at the bottom of the buccal flap to enhance its elasticity and to achieve tension-free primary closure, the tumor cavity was packed with absorbable gelatin sponge (Gelfoam®, Pfizer, New York, USA), and the interrupted sutures were placed (4-0 Vicryl, Johnson & Johnson Medical, Norderstedt, Germany) (Figure 6(e)).

The surgical specimen was submitted for histopathological examination, and the diagnosis confirmed the previous incisional biopsy (Figure 6(f)).

Healing was uneventful without any complications and follow-up was performed at 3, 6, and 12 months.

**Figure 7:**
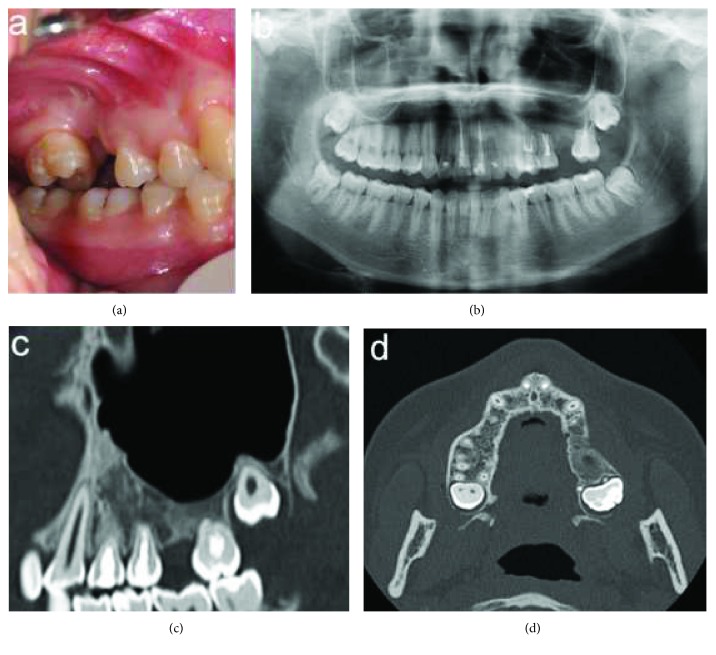
Twelve-month follow-up: (a) intraoral view, (b) ortopantomography, (c) CT sagittal view, and (d) CT axial view.

One year after the surgery, clinical examination and radiographs showed restitutio ad integrum both of the bone and the soft tissues and no local recurrence (Figure 7).

## 3. Discussion

An extrafollicular case of AOT in the posterior maxillary region was described, as suggested in the review by Philipsen et al. [2]. The authors recommended to discontinue reporting classical follicular cases but to continue reporting well-documented cases of the extrafollicular variant, with indication of the exact position.

AOT is relatively rare, with only 1558 cases reported in a total of 436 publications retrieved in a recent updated analysis of the literature undertaken without time restrictions up to July 2018 [6].

AOT affects more frequently the second and third decade of life (range: 1-83 years), female patients (male : female ratio 1 : 1.9), and maxilla over the mandible (ratio: 2.1 : 1), with a preference for the anterior portion of the jaws [1, 2, 5–7].

AOT has three distinct clinicopathological variants without any relevant clinical and radiographic differences between them: intraosseous follicular (73% of all cases), related to retained tooth, most often a canine; intraosseous extrafollicular (24%), which is subdivided into E1 no relation to tooth structure either erupted or unerupted, E2 intraradicular adjacent roots, E3 superimposed on the root apex, and E4 superimposed on the midroot level; and peripheral (3%), in the soft tissue overlying the tooth-bearing area [5, 6, 8–10].

For the follicular variant, there is histologic and immunohistochemical evidence that it arises from the reduced enamel epithelium of the dental follicle, whereas origin of the extrafollicular variant is less clear [11, 12]. Philipsen et al. [2] argued that the identical histology in all AOT variants points towards a common origin and implicate the dental lamina or its remains.

The lesion ranges from 2 to 7 cm in size, has a slow growing pattern which can result in a painless expansion of the jaws, and often leads to the displacement of the adjacent teeth, whereas root resorption is more rare [6, 10]. The aggressive behavior of the AOT is quite exceptional, with only a few cases reported in the literature, mainly in the mandible [13].

In this report sex, age, and clinical and histopathologic appearance were consistent with the literature, but the location in the posterior region of the maxillary jaw and some features, such as root resorption, cortical perforation, and small radiopaque foci inside the radiolucent lesions, were less frequent.

Conservative surgical approach with complete enucleation of the lesion is the treatment of choice, justified by the benign behavior, presence of clear demarcation, and low rates of recurrence [6, 14].

In the present case, in addition to excision of the lesion, surgical management required the extraction of the first upper molar, mesial root amputation of the second molar, and apicectomy of the premolars and the remaining roots of the second molar.

## 4. Conclusion

The present case was reported to increase the knowledge about morphological characteristics, clinical course, radiographic and histopathological features, and surgical therapy of an extrafollicular AOT arising in the posterior maxilla.
